# Patient recall of postoperative protocols following hand surgery does not differ by information provider: a randomized clinical trial

**DOI:** 10.3389/fsurg.2025.1559161

**Published:** 2025-05-30

**Authors:** Dhruv Mendiratta, Rohan Singh, Aleksandra McGrath

**Affiliations:** ^1^ Department of Orthopaedics, New Jersey Medical School, Rutgers, The State University of New Jersey, Newark, NJ, United States; ^2^ Department of Clinical Science, Faculty of Medicine, Umeå University, Umeå, Västerbotten, Sweden; ^3^ Department of Surgical and Perioperative Sciences, Faculty of Medicine, Umeå University, Umeå, Västerbotten, Sweden

**Keywords:** patient care, postoperative recall, healthcare literacy, hand surgery, clinical trial

## Abstract

Understanding of postoperative care is limited in patients who undergo ambulatory surgery. This study compares patients' recall of information regarding postoperative self-care when being verbally informed by either a surgeon or assistant nurse postoperatively prior to discharge. Secondary objectives for this study are to compare differences in patients' level of “feeling that they understood the information”, stress, and satisfaction. A non-blinded randomized single-center controlled trial was conducted at a hand surgical unit in Northern Sweden (Trial Registration ID: NCT03893968). Patients were randomized into a control (surgeon) or intervention group (assistant nurse). Patients were asked seven questions about postoperative self-care one week postoperatively via telephone call, yielding a maximum score of seven points. Thirty-nine patients were informed by assistant nurses, and thirty-three patients were informed by surgeons. There was no difference in recall between the two groups (4.95 vs. 5.15, *p* = 0.5). Patients from both groups lacked knowledge on postoperative outcomes (41.0% vs. 42.4%). The mean scores for “feeling of having understood the information” (mean of 9.23 for patients informed by assistant nurses vs. mean of 9.45 for patients informed by surgeons) and satisfaction (9.69 vs. 9.45, respectively) was high, while mean scores for stress was low (1.38 vs. 1.18, respectively). Few patients answered all questions correctly: 8.3% of the patients answered all questions correctly, and 37.5% of the patients answered six or more questions correctly. The findings suggest that surgeons and assistant nurses are equally good at verbally informing patients regarding postoperative hand-surgical self-care. More effort is needed to make patients understand symptoms of postoperative infections.

## Introduction

Healthcare professionals' communication of information is key to making patients understand their diagnosis, treatment, and prognosis (1, 2). Despite this, the comprehension of medical information is generally poor. Kriwanek et al. found in 200 patients undergoing laparoscopic cholecystectomy that 49% had insufficient understanding of the procedure, and 69% could not name a single possible complication (3). Lack of patients' knowledge after being informed has been shown after consenting for other surgical procedures and in emergency department care (4–9). Making patients understand and recall discharge information is of importance as it increases patients' ability and confidence in managing their own health condition, while the opposite leads to patients accessing health facilities or mismanaging their self-care (10, 11).

Today ambulatory surgery, also called day surgery, stands for about 60% of the surgical procedures in Sweden, measuring to over a million procedures per year (12, 13). Ambulatory surgical patients arrive at the hospital, undergo surgery, and are discharged from the hospital within the same day. Whereas inpatient surgical patients receive care on the ward during the postoperative period, patients in day surgery care must understand and solely manage their care (14, 15). Even though patients often express confidence in managing their care before discharge, many come to realize that their actual understanding of the postoperative care required is insufficient (11, 14, 16, 17). A gold standard regarding how to inform patients to ensure understanding of information has not yet been identified (9, 18).

Previous studies indicate that although patients are generally equally satisfied, patients seem to prefer doctors for medical aspects of care and other healthcare-professions for educational aspects (19, 20). A Swedish qualitative study showed that information provided by nurses and assistant nurses were in comparison to doctors easier to understand, as they took more time and used less medical terminology when explaining (19). Meta-analyses from primary and preoperative healthcare, regarding substitutions of doctors by other healthcare-professions, have shown that there is not enough evidence to permit assessment of differences in outcomes (ex. complications, satisfaction) (21, 22). Although some research indicates that other health-professionals are easier to understand compared to doctors (19, 20), the authors have not identified any quantitative studies verifying this. Based on these findings, the hypothesis of this study was that other healthcare-professionals are better at educating patients than surgeons.

The objective of this study is to compare differences in recall of discharge information regarding postoperative self-care in ambulatory hand-surgical patients, when being informed by either a surgeon or an assistant nurse. Secondary objectives for this study are to compare differences in patients' level of “feeling that they understood the information”, stress, and satisfaction, and to compare differences in the number of healthcare contacts initiated by patients because of not recalling or understanding the postoperative information.

## Methods

### Study design

This was a single-center, non-blinded, randomized controlled trial. Patients scheduled to undergo ambulatory hand-surgery were randomized to either being verbally informed by the operating surgeon or by an assistant nurse regarding their postoperative self-care postoperatively prior to discharge. Of the two parallel groups, the control group was patients informed by surgeons and the intervention group was patients informed by assistant nurses. The allocation ratio was 1:1. The study was set to identify superiority in the intervention group. Patients were subsequently contacted one week postoperatively after discharge via telephone call to assess instruction recall. This study was approved by the regional ethical review board. This study is registered with ClinicalTrials.gov (ID: NCT03893968).

### Participants

Included were ambulatory hand-surgical patients aged eighteen or older about to undergo surgery under local anesthetics. Patients were excluded if they could not speak Swedish or were diagnosed with a disease associated with cognitive impairment (e.g., dementia). Patients were recruited during nine consecutive weeks in 2018. Written and oral consent for the study was obtained on arrival to the unit the day of the surgery.

### Study setting

The study was conducted within the hand-surgical unit in Sweden. The hand-surgery unit serves both the local population and is a tertiary referral center. There were a total of seven surgeons and seven assistant nurses participating in the study, all having several years of experience working with hand-surgical care. Prior to the study, doctors had the formal responsibility of informing patients about their postoperative care. However, despite it being the surgeons' responsibility, the task of informing patients was at times performed by assistant nurses. After receiving the information, patients were discharged and left the clinic. Normally patients receive complementary written information after being informed verbally. Patients included in the study did not receive the written information, since it might have been a confounding factor in the understanding and recalling of information (18, 23–25).

### Randomization

Determination of which healthcare-profession would be informing patients was done following a single randomization procedure. No blocking or stratification was used. Randomization was performed using an envelope containing one-hundred and ten notes with fifty-five notes each for both alternatives. Due to the simple randomization procedure, the allocation sequence was not possible to foresee until the interventions were assigned. Authors were responsible for recruiting, randomizing patients, and collecting the data.

### Information regarding postoperative self-care

After the surgery and before discharge, patients were informed by either the operating surgeon or the assistant nurse. The information focused on postoperative self-care, such as the use of over-the-counter medications, the importance of elevating the hand and examples of when to contact the unit after the surgery. Patients received the same standardized information, regardless of the type of ambulatory hand-surgical procedure they underwent. Both elective and emergency procedures were included.

The surgeons and assistant nurses were instructed to use the, normally given, complementary written information as a checklist (Supplementary Appendix 1) when informing patients. The surgeons and assistant nurses could bring the sheet with them to the counseling session. They were not allowed to give patients a copy of the sheet or to show the sheet to the patients. The participants did not know which aspects of self-care information would be tested by the interviewer.

### Telephone interview

Telephone interviews were performed seven days after the surgery using a structured questionnaire (Supplementary Appendix 2). The questionnaire included seven questions regarding patients' characteristics. For the main objective, seven questions were asked testing patients recall/knowledge of their postoperative self-care. Each question evaluated the patients' knowledge of a unique aspect of postoperative self-care.

For the last three questions, the patients could rate on a 1–10 scale their level of “feeling of having understood the information”, stress, and satisfaction (secondary objective). “Feeling of having understood the information” was explained to patients as “perception of having fully understood all the information and knowing fully what to do when leaving the hospital”. For assessing “stress”, the interviewer asked about the level of stress that the patient had experienced during the week following the surgery. Regarding “satisfaction”, the interviewer asked and assessed how satisfied the patients were with the way the personnel had informed the patient (e.g., not used difficult or confusing language, had given the patient time to ask questions). Lastly, patients were asked some open-ended questions regarding the information they received, including what in their opinion hindered understanding of information.

### Review of patients' journal

Patients' notes were reviewed one month after the surgery to check if the patient had contacted any healthcare-providers due to not having understood/recalled information (secondary objective). All type of contact (e.g., telephone-call etc.) initiated by the patient because of not understanding or being unable to recall the information was noted (e.g., any complication caused by not adhering to instructions, having to contact healthcare for reassurance regarding how to manage postoperative self-care).

### Assessment of patient recall

For assessment of patient recall, each correctly answered question regarding postoperative self-care was valued as one point, meaning that patients answering all seven questions had a total of seven points. The primary endpoint regarding patient recall was achieving one point better (one more correctly answered question) than the other healthcare-profession.

### Sample size

Sample size calculation (two-sided equality) showed that to detect a difference of one point for primary objective (one more correctly answered question), with a standard deviation (SD) of 1.25, a statistical power of 80%, and 5% significance level, a total of sixty-four patients had to be recruited to the study.

### Statistical analysis

Data were analyzed using IBM SPSS for Windows, Version 25.0 (IBM Corp. Released 2017. Armonk, NY, USA). As assumption of normality could not be met, Mann–Whitney *U* test was used for analyzing for the primary endpoint regarding information recall. Mann–Whitney U was also used for analyzing the secondary objectives of patents “feeling of having understood the information”, stress, and satisfaction. As such, medians and interquartile ranges (IQR) were reported for nominal variable comparison between the two groups. However, in understanding the data practically, means are reported for a more robust comparison. Effect sizes (r) are reported to determine practical application of the data with values closer to one being more meaningful. Chi-square test was used for calculating the significance in differences in proportions of correct answers for each separate question, [e.g., the number of patients answering correctly on question 1 in the intervention- and control-group (Table 1)]. Significance was achieved by a *p*-value less than 0.05.

**Table 1:** Distribution of correct responses by question.

Informant group	Patients informed by assistant nurses		Patients informed by doctors		Total			
	(*n* = 39)		(*n* = 33)		(*n* = 72)			
	*N*	%	*N*	%	*n*	%	*p*-value	Phi
Question 1: “Should the hand be held in any specific position?” “Correct answer: Yes, in an elevated position.”								
Incorrect answer	3	(7.7)	2	(6.1)	5	(6.9)		
Correct answer	36	(92.3)	31	(93.9)	67	(93.1)	*p* = *	
Question 2: “Was it recommended to move your fingers?” “Correct answer: Yes.”								
Incorrect answer	7	(17.9)	6	(18.2)	13	(18.1)		
Correct answer	32	(82.1)	27	(81.8)	59	(81.9)	*p* = 0.980	−0.003
Question 3: “Should your train the hand/do any specific movement?” “Correct answer: Yes, flexion and extension. Regularly.”								
Incorrect answer	10	(25.6)	6	(18.2)	16	(22.2)		
Correct answer	29	(74.4)	27	(81.8)	56	(77.8)	*p* = 0.448	0.089
Question 4: “How much can you use your operated hand?” “Correct answer: Not fully, holding a light object is ok.”								
Incorrect answer	20	(51.3)	10	(30.3)	30	(41.7)		
Correct answer	19	(48.7)	23	(69.7)	42	(58.3)	*p* = 0.072	0.212
Question 5: “Is it recommended to use a sling?” “Correct answer: No.”								
Incorrect answer	15	(38.5)	16	(48.5)	31	(43.1)		
Correct answer	24	(61.5)	17	(51.5)	41	(56.9)	*p* = 0.392	−0.101
Question 6: “Should any special measure be taken when showering?” Correct answer: Have the hand in a plastic bag (keep the dressing dry).								
Incorrect answer	2	(5.1)	2	(6.1)	4	(5.6)		
Correct answer	37	(94.9)	31	(93.9)	68	(94.4)	*p* = *	
Question 7: “Name three symptoms of a postoperative infection.” “Possible correct answers: Pus, pain, redness, swelling, fever.”								
Incorrect answer	23	(59.0)	19	(57.6)	42	(58.3)		
Correct answer	16	(41.0)	14	(42.4)	30	(41.7)	*p* = 0.905	0.14

Significance calculated using Chi-Square Test.

* Chi-Square Test not possible due to 2 cells (50%) having expected count less than 5.

### Ethics statement

This study was approved by the regional ethical review board. Any patient, service user, or participant (or that person's parent or legal guardian) in any research, experiment, or clinical trial described in our paper has given written consent to the inclusion of material pertaining to themselves, that they acknowledge that they cannot be identified via the paper; and we have fully anonymized them.

## Results

### Participants

**Figure 1:**
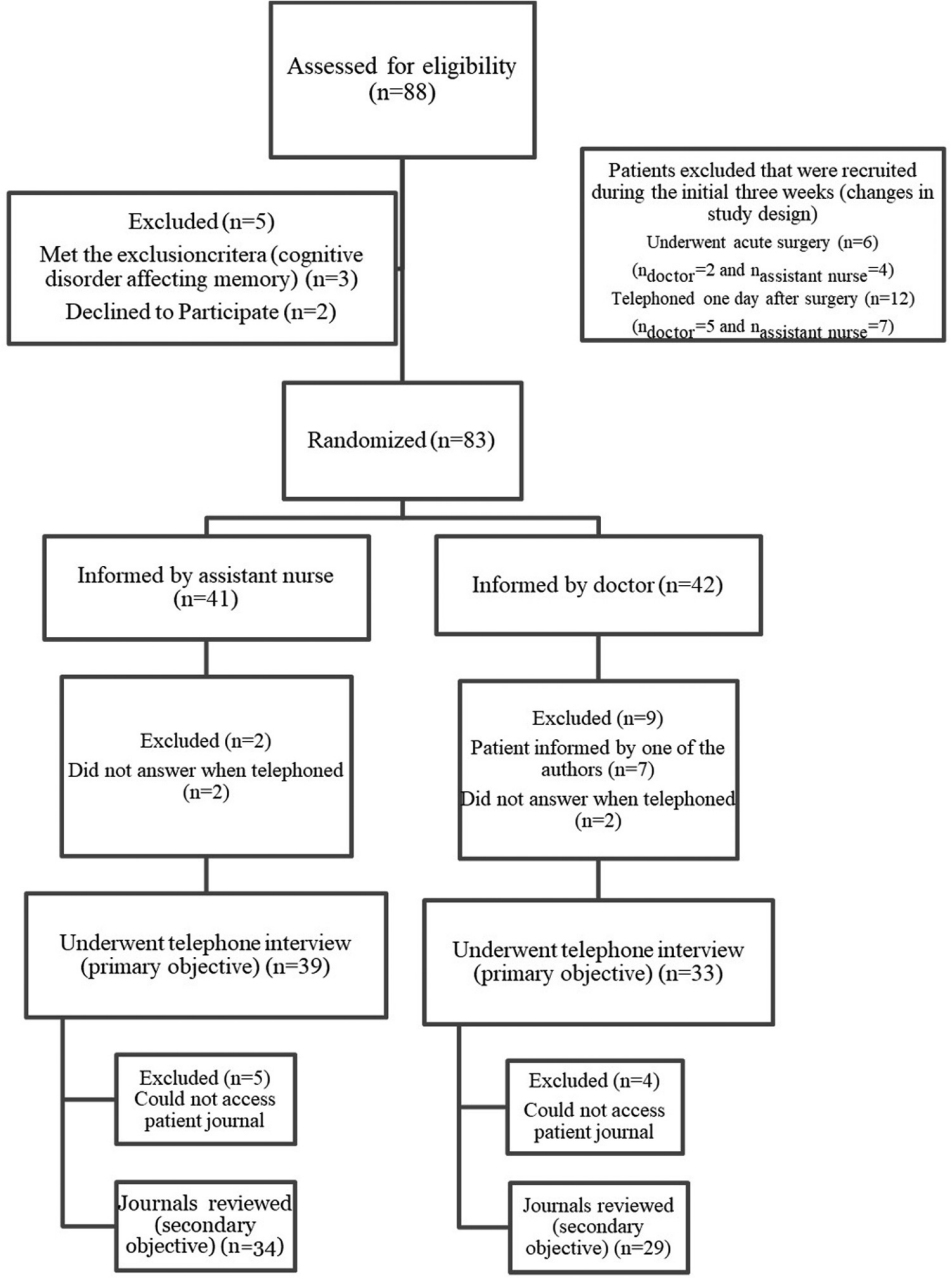
Patient flow-chart.

The number of eligible patients recruited was eighty-three. Seventy-two patients underwent the telephone interview, and sixty-three patient journals were accessible and reviewed (Figure 1). The participants' age ranged from nineteen to eighty-six years. The mean age of the patients informed by assistant nurses was 55.8 years (SD = 19.5) and for the patients informed by doctors 59.5 years (SD = 16.6).

The baseline characteristics of the patients telephoned in the study showed that somewhat more patients in the group informed by assistant nurses had not undergone surgery previously (20% for assistant nurses compared to 12% for surgeons). Apart from this, the characteristics of the groups were similar (Table 2). Majority of the patients have previously undergone surgery (either within the upper extremity or elsewhere). Seven patients were prescribed antibiotics preventively on the day of surgery.

**Table 2:** Patient characteristics by informant group.

Informant group		Patients informed by assistant nurses		Patients informed by doctors		Total	
		(*n* = 39)		(*n* = 33)		(*n* = 72)	
		*N*	%	*N*	%	*N*	%
Gender	Male	16	(41.0)	13	(39.4)	29	(40.3)
	Female	23	(59.0)	20	(60.6)	43	(59.7)
	Other	0		0		0	
Age	18–34	7	(17.9)	2	(6.1)	9	(12.5)
	35–54	9	(23.1)	10	(30.3)	19	(26.4)
	55–74	17	(43.6)	14	(42.4)	31	(43.05)
	75+	6	(15.4)	7	(21.2)	13	(18.05)
Education	No education	0		0		0	
	0–8 years primaryschool	6	(15.4)	3	(9.1)	9	(12.5)
	9–10 years primaryschool	3	(7.7)	4	(12.1)	7	(9.7)
	1–2 years at gymnasium	8	(20.5)	6	(18.2)	14	(19.4)
	>2 years at gymnasium	13	(33.3)	9	(27.3)	22	(30.6)
	1–3 years at university	5	(12.8)	7	(21.2)	12	(16.7)
	>3 years at university	4	(10.3)	4	(12.1)	8	(11.1)
	Ph.D-education	0		0		0	
	Other	0		0		0	
Native language	Swedish is native language	37	(94.9)	33	(100)	70	(97.2)
	Swedish is a secondary language	2	(5.1)	0	(0)	2	(2.8)
Language skills	Speaks fluently Swedish	37	(94.9)	33	(100)	70	(97.2)
	Speaks Swedish well but not fluently	2	(5.1)	0	(0)	2	(2.8)
Prior healthcare experience	Had underwent surgery of arm/hand	16	(41.0)	15	(45.5)	31	(43.1)
	Had underwent surgery (any part of body)	31	(79.5)	29	(87.9)	60	(83.3)
	Have had a plaster cast	14	(35.9)	11	(33.3)	25	(34.7)
	Have had sutures	30	(76.9)	29	(87.9)	59	(81.9)
	Have had a wound-infection	7	(17.9)	5	(15.2)	12	(16.7)
	Had not undergone any prior surgery	8	(20.5)	4	(12.1)	12	(16.7)
Profession	Have worked in surgical healthcare	1	(2.55)	2	(6.1)	3	(4.2)
	Have worked in non-surgical healthcare	4	(10.25)	1	(3.0)	5	(6.9)
	Have not worked within healthcare	34	(87.2)	30	(90.9)	64	(88.9)
Antibiotic counseling	Received antibiotics preventative regarding postoperative infection	3	(7.7)	4	(12.1)	7	(9.7)

### Measured recall of information

Comparing the questions separately, no significant difference was found between the groups (Table 1). Despite not being significantly different, a higher number of patients informed by surgeons compared to assistant nurses answered correctly the question concerning how much functional usage is appropriate for the operated hand (69.7% compared to 48.7% respectively, *p* = 0.072).

The lowest number of patients answering correctly was found for the question regarding symptoms of postoperative infection; within the entire cohort, only 41.7% of patients answered this question correctly and were able to name three symptoms of postoperative infection. 52.8% of patients could name two symptoms, and 66.7% of patients could name only one symptom (no significant difference between groups informed by surgeons and assistant nurses). The other two questions where the patients scored lower in general were questions about the sling (57% answered correctly) and the previously mentioned question about the appropriate functional usage (58.3% answered correctly).

**Figure 2:**
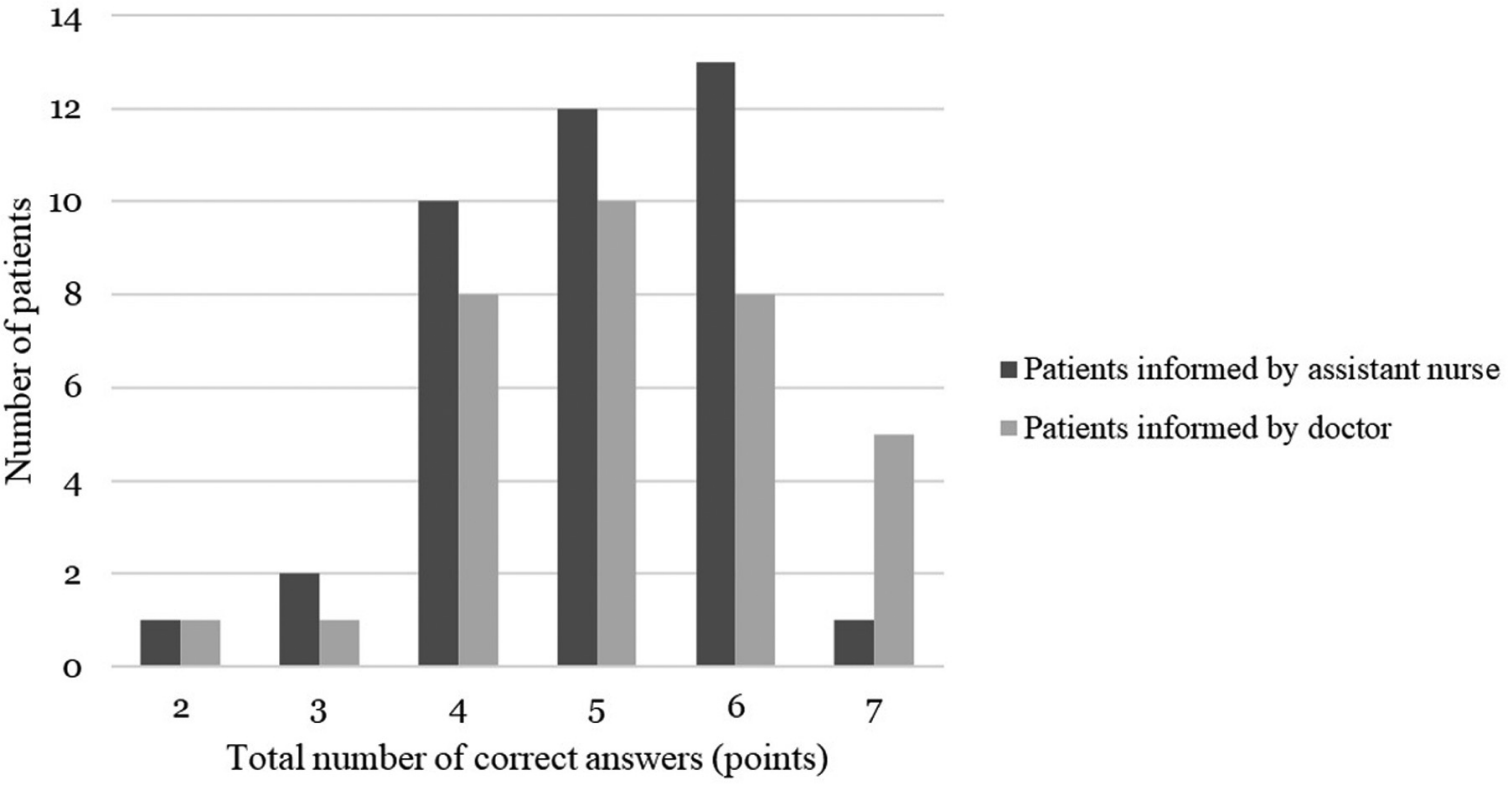
Total amount of correct answers per patient (maximum seven).

Most patients answered four to six questions correctly (84.7%) (Figure 2). Patients informed by surgeons more often answered correctly on all seven questions (15.2% vs. 2.6%, *p*-value not calculable with chi-square due to 2 squares with an expected value less than 5).

Regarding the total amount of correct answers, no significant difference could be seen between the groups (Table 3).

**Table 3:** Total amount of correct responses (maximum seven).

Informant group	Mean	Standard deviation (SD)	Median	IQR	*p*-value	Effect size (r)
Patients informed by assistant nurses	4.95	1.07	5	4–6		
Patients informed by doctors	5.15	1.23	5	4–6	*P* = 0.50	−0.08

Significance calculated using Mann–Whitney *U* test: *U* = 586, *p* = 0.501, *r* = −0.08.

### Secondary objectives

No significant differences could be found in either of the measured secondary outcomes between the groups (Table 4). The mean scores for “feeling of having understood the information” (mean of 9.23 for patients informed by assistant nurses vs. mean of 9.45 for patients informed by surgeons) and satisfaction (9.69 vs. 9.45, respectively) was high, while mean scores for stress was low (1.38 vs. 1.18, respectively). The majority of the patients gave the maximum score (10 out of 10) on “feeling of having understood the information” (70.8%), even though few patients answered all questions correctly (8.3% of the patients answered all questions correctly and 37.5% of the patients answered six or more questions correctly).

**Table 4:** Secondary outcomes: rated on a 1 to 10 scale.

Informant group	Patients informed by assistant nurses (*n* = 39)			Patients informed by doctors (*n* = 33)		Post	*p*-value	Effect size (r)
	Mean	Standard deviation (SD)	Median [IQR]	Mean	Standard deviation (SD)	Median [IQR]		
Feeling of having understood the information*	9.23	1.25	10 [9–10]	9.45	1.30	10 [10–10]	*p* = 0.202	−0.15
Stress**	1.38	1.14	1 [1–1]	1.18	0.53	1 [1–1]	*p* = 0.638	−0.06
Satisfaction***	9.69	0.86	10 [10–10]	9.45	1.43	10 [10–10]	*p* = 0.689	−0.05

Significance calculated using Mann–Whitney *U* test.

* *U* = 553.

** *U* = 618.5.

*** *U* = 620.5.

The review of the patients' journals revealed that one out of the sixty-three patients (informed by a surgeon) had established contact with health care due to the undergone surgery. The reason was pain in the operated area where the patient was unsure of the amount of pain that was normal postoperatively.

When the patients were asked open-ended questions regarding factors which hinder or facilitate understanding of the information, most patients reported that it was contextual factors that they felt had mattered. The most reported comment was that the patients felt that assistant nurses or surgeons acted as if they were stressed that influenced their understanding negatively. The patients reporting this observation experienced that the stress affected them in turn. They reported experiencing that the healthcare professional did not explain the information in detail due to shortage of time (perceived by patients), which led to the patients not wanting to ask additional questions and therefore taking up more time. This instance was reported in 20% of cases and equally common in both groups.

## Discussion

Patients generally report satisfaction with information delivered by the nursing staff due to ease of understanding and relatability when compared to physicians (20). Thus, in surgical centers, assistant nurses play a vital role in the delivery of patient-centered care in the perioperative and postoperative settings by both monitoring the patient and delivering information on post-discharge protocols (26, 27). In this study, we systematically examined if the retention of postoperative information differed based on the information provider. Data from this study showed no difference in the total number of correct answers by provider. Despite prevailing beliefs that patients prefer the holistic care provided by the nursing staff, we found that surgeons and assistant nurses may be equal at making patients understand and recall postoperative information (28). This study is the first to develop a quantitative assessment for the difference in information delivery between these two professions. Although a vast difference between the education between a surgeon and an assistant nurse exists, neither is superior to the other in delivering key postoperative information to the patient. Reasons for this may include the individual strengths and weaknesses of each of these professions that when taken together could make for a superior educator. In support of this theory is the finding that patients informed by surgeons more often answered correctly regarding the functional usage of the operated hand, although no significant difference was found. A major critique for physicians has been the dearth of perceived time they may have for the patients, discouraging further clarification. Dugdale et al. posit that the lack of time is implicated in affecting components of care, such as patient satisfaction and outcomes (29). In our study, patients had perceived both surgeons and assistant nurses to have a shortage of time equally.

Especially following hand surgery, understanding the postoperative protocols are of great importance, as adherence to them can prevent unscheduled postoperative encounters and complications, such as infection. In turn, this can prevent unnecessary utilization of resources by the healthcare system. Even though patients felt that they fully understood the information given (mean 9.33 out of 10), few had full knowledge of the different aspects of postoperative self-care. This finding indicates that patients might overestimate their actual level of knowledge of their medical condition, a problem previously identified in the literature (11, 14, 16, 17). In light of our findings, the gold standard should be to provide written information to patients, as verbal information is poorly retained; Dhellemmes et al. purported a similar finding in patients that are discharged following hand surgery at a trauma center in France (30). Both our study and theirs call for better patient understanding via standardized information that is adaptable to each patient and each injury.

Menendez et al. found that emergency department visits following hand surgery are usually related to pain or wound issues (31). Thus, the finding that many patients were unaware of the symptoms of infection in the postoperative setting bodes poorly on the identification of infection development and wound care. Further, following surgery, early mobilization of the hand is critical in preventing postoperative stiffness and need for reoperation. Accordingly, understanding the function of dynamic hand motions during the rehabilitation period is crucial, as it portends recovered hand function (32). Fortunately, in our study most patients knew the range of hand movement that was necessitated.

The findings from this study should be interpreted with caution. The most obvious source of an eventual bias is the study being non-blinded, probably affecting both groups trying to perform better than usual. As previously mentioned, patients participating in the study did not receive the standard written information. Both above-mentioned factors mean that the normal situation was not the one studied. The study was set in a hand-surgical setting and a single-center study, meaning that the generalizability of the study is questionable. The sample size calculations were performed for seven questions with the total score of seven points as primary objective, making it hard to interpret differences in the number of correct answers in the separate questions (Table 1). Still, for investigating the primary objective in regard to the primary endpoint, the number of patients recruited is sufficient.

This paper is, despite its limitations, a first step in gaining quantitative knowledge of the differences between healthcare professionals as educators. More research is recommended, preferably in a blinded study and in other healthcare settings.

## Conclusions

Data from this study showed no significant difference in the total amount of correct answers between the groups informed by surgeons and assistant nurses. The data imply that the recall of information was equally good in the two groups in a non-blinded ambulatory hand-surgical setting. The number of patients having knowledge of the symptoms of postoperative infections was low. While patients felt that they had fully understood the information received, few had full knowledge of the information. The findings indicate that surgeons and assistant nurses are equally good as educators when informing patients regarding postoperative hand-surgical self-care. More effort must be put to make patients understand/recall symptoms of postoperative infections.

## Data Availability

The raw data supporting the conclusions of this article will be made available by the authors, without undue reservation.
