# Supplementary material for: Patient recall of postoperative protocols following hand surgery does not differ by information provider: a randomized clinical trial
Source: Front Surg. 2025 May 30;12:1559161. doi: 10.3389/fsurg.2025.1559161 (PMC12162562; doi:10.3389/fsurg.2025.1559161)
Supplement: Supplementary file 1 [file Table5.docx]

Appendix 1

Appendix 1) Check-list (Translated from Swedish to English)

Patient information/checklist for informing patients regarding postoperative self-care

After your surgery

After the surgery, the pressure of the tourniquet is released. The local anesthetic gradually wears off and after 5-10 minutes you’ll regain normal sensation in your arm. Sometimes anesthetics with extra-long duration is used, in this case, it takes more time before the sensation in your arm/hand is back.

Dressing

After a hand surgery, it is extra important to have your hand in an elevated position, at the height of your shoulder for 3-4 days. At night time you can use an extra pillow under your hand. The elevation is important to avoid swelling of the arm/hand. We do not recommend the use of a sling, due to the risk of getting stiff in the neck and shoulder. If the bandage/cast is too tight, despite having the hand in an elevated position, it is sometimes necessary to open the bandage/cast. In this case, contact a medical healthcare provider immediately!

The dressing and cast shall be kept dry. When showering, put a plastic bag over the dressing/cast and attach it with tape or a rubber band. Place your hand so it does not get in touch with the water. It takes one day before the cast is completely solidified. Be extra careful during the first 24 hours.

Training of your hand

If you have not received any other instructions from your doctor, it is recommended to move your fingers every day, a couple times a day. Bend and stretch/extend your fingers all the way, stretch you operated arm up in the air above your shoulder and pump a couple of times with the hand to avoid swelling. Apart from that, you should let the operated arm rest. You might hold a telephone or a newspaper in your hand but should not use your hand more than that. Remember to NOT support yourself on the operated hand.

Sutures

The sutures are usually taken by your primary care nurse if you have not received any other instructions from your doctor. You will be given written information where it is described when and how the sutures should be removed. You must contact the primary care nurse yourself, preferably immediately on the day of your surgery. Take the written information with you when you go there. You will also be given a form that the nurse should fill in if there are signs of an infection when the sutures are removed. After the sutures have been removed, you might experience some pain and the scar might be red, this should gradually diminish.

Pain

The first days after the surgery, you will experience pain around the wound. Regarding the pain, paracetamol (not more than 1 gram taken 4 times a day) is usually sufficient. If you are allowed to take ibuprofen or a similar medicine, you can take it in combination with paracetamol according to the patient information leaflet on the packaging. When appropriate, you will receive from us some stronger painkillers, which are sufficient for 2-5 days after the surgery, when most patients need painkillers the most.

Infection

After your hand-surgery, your hand is going to feel better every day. If you have an infection, this pattern changes; it starts to hurt more after that the pain at first lessened.

The pain from the infection most often starts three days after the surgery. If you do experience more pain in and around the wound, you should immediately contact health-care provider (us or a primary care provider nearest to you). It is important that a doctor looks at the wound on the same day you become worried that you might have an infection!

Other signs of infection are:

- Pain that starts a couple of days after the surgery, which increases over time. The pain can be throbbing, especially around the area you were operated at.
- A red zone around the wound which grows (a couple of millimeters of redness around the edges of the wound and the stitches are a normal reaction to healing).
- Swelling in the area around the wound which increases.
- Fever.
