# Supplementary material for: Patient recall of postoperative protocols following hand surgery does not differ by information provider: a randomized clinical trial
Source: Front Surg. 2025 May 30;12:1559161. doi: 10.3389/fsurg.2025.1559161 (PMC12162562; doi:10.3389/fsurg.2025.1559161)
Supplement: Supplementary file 2 [file Table6.docx]

Appendix 2) Questionnaire (Translated from Swedish to English)

**Questionnaire**

**PART 1: BACKGROUND-QUESTIONS**

**Question 1: How old are you?**

- 18 – 29 years old
- 30 – 39 y/o
- 40 – 49 y/o
- 50 – 59 y/o
- 60 – 69 y/o
- 70 – 79 y/o
- 80 – 89 y/o
- 90 years old or older

**Question 2: What gender do you identify yourself as?**

- Male
- Female
- Other

[Control][Control]

**Question 3: Which is the highest level of education you have completed?**

- No education
- 0-8 years primary school
- 9-10years primary school
- 1-2 years at gymnasium (equivalent of high school)
- >2 years at gymnasium
- 1-3 years at University
- > 3 years at University
- Ph.D-education (Doctorate)
- Other
- Don’t know

[Control]

**Question 4: Is Swedish your native language?**

Clarifying: The native language is the language that a person has been exposed to from birth and became your first spoken language

- Swedish is my native language
- Swedish is my secondary language
- Don’t know

[Control]

**Question 5: How good is your ability to speak Swedish?** [Control]

- I can speak Swedish fluently and excellent.[Control]
- I can speak Swedish very well. I might need help to understand some difficult words.[Control]
- I can speak Swedish well. I might need help to understand words. [Control]
- I can speak some Swedish. I often need help to understand what others are saying.
- I can only speak very little Swedish. I need help a lot of help to be anything that is being said.

[Control]

**Question 6: Do you work/have worked within Healthcare at a hospital or otherwise?**

Clarifying: “Administrative work or working with dentistry does not apply to this question.”

[Control]

- YES. I work/have worked within healthcare at a surgical department or at an E.R. (emergency department).[Control]
- YES. I work/have worked within healthcare, but not at a surgical department or at an E.R.
- NO. I have not worked in healthcare.

**Question 7**[Control]**: Here comes a few statements regarding prior surgery.**

Clarifying: “Following statements are asked in question form: E.g. “have you ever undergone surgery of your arm/hand?”. The questions are answered with yes/no-answers.”[Control]

- I have undergone surgery on my arm/hand.[Control]
- I have undergone surgery (another part than arm/hand)[Control]
- I have had a cast prior to this surgery.
- I have had stitches prior to this surgery.[Control]
- I have had a wound infection. [Control]
- I have NOT undergone any type of surgery prior to this surgery-.

**PART 2: ASSESMENT OF COMPREHENSION/RECALL**

**Question 8: Shall the hand be held in any specific position after the surgery?**

Correct answer: The hand should be held at the height of the shoulder/above the heart (1p)

**Question 9: Are you allowed to move your fingers after the surgery?**

Correct answer: Yes! (1p).

**Question 10: Should you exercise with your hand in any specific way?**

Correct answer: The fingers shall be bent and extended (flexion/extension) (1p).

**Question 11: How much are you allowed to use the operated hand?**

N.b. A verbatim answer is not required for a point here. The important here is that the patient has understood that he/she is not allowed to use the hand fully.

Correct answer: Not fully, to hold a telephone or newspaper is fine, but not to lift heavy objects (1p).

**Question 12: Is it recommended to use a sling?**

Clarifying:” A sling is a strap/bandage used to support the injured arm”.

Correct answer: No (1p).

**Question 13: Should you take any special precaution regarding showering the first day?** Correct answer: A plastic bag shall be used over the injured arm/hand (1p).

**Question 14: Name three symptoms that are present in a wound infection.**

Correct answer (3 correct answers gives 1p): Pain, heat, redness, swelling, fever.

**PART 3: LEVEL OF CONTROL/STRESS/SATISFACTION**

Clarification by the interviewer: “It sometimes happens that patients feel stress or anxiety after the surgery, due to the feeling of insecure regarding what to do after the surgery. E.g. how to train the arm, how to manage the wound etc. The following questions are regarding if you have felt stress due to the feeling of uncertainty regarding what to do after the surgery.”

” The following questions are answered on a scale from 1-10 where 1 is the lowest and 10 the highest.”

**Question 15: Did you feel that you had understood what to do after the surgery?** Clarification: What we specifically want to know if you felt this on the day of your surgery, at the moment you left the hospital. “Rate your answer on a scale from 1-10, where 1 is the lowest score (meaning that you felt that you didn’t understand at all what to do) and 10 the highest score (meaning that you felt that you fully understood what to do after the surgery).”

1---------------------------------------------------10.

[Control]

**Question 16: Have you experienced stress after the surgery?**

Clarification:” Rate your answer on a scale from 1-10 where 1 is the lowest score (no stress at all) and 10 is the highest (severely stressed)”

1---------------------------------------------------10.

**Question 17: Have you felt anxious after the surgery?**

Clarification:” Rate your answer on a scale from 1-10 where 1 is the lowest score (no anxiety) and 10 is the highest (severely anxiety)”

1---------------------------------------------------10.

[Control]

**Question 18: Are you satisfied with the way you have been informed?**

Clarification:” Rate your answer on a scale from 1-10 where 1 is the lowest score (not being satisfied at all) and 10 is the highest (feeling very satisfied)”

1---------------------------------------------------10.

**OPEN-ENDED QUESTIONS**

The interviewer ends the by asking some open-ended questions, usually by follow-up questions for clarification regarding previously asked questions. After that, the interviewer ends by asking some final open-ended questions.

Was something easy/hard to understand?

Did the staff do something specific that led to the information becoming easy/hard to understand?

Had you wanted them to do something different?

What do you think might have affected how much you remember?

What do you think made you follow/not follow the recommendations given?

How many days did you have your hand in an elevated position?

How many days after surgery did you move your fingers regularly?

Any other thoughts?
